# Current Status of Phage Therapy against Infectious Diseases and Potential Application beyond Infectious Diseases

**DOI:** 10.1155/2022/4913146

**Published:** 2022-10-03

**Authors:** Hao-Ming Xu, Wen-Min Xu, Long Zhang

**Affiliations:** ^1^Department of Gastroenterology and Hepatology, Guangzhou Digestive Disease Center, Guangzhou First People's Hospital, School of Medicine, South China University of Technology, Guangzhou 510180, China; ^2^Department of Endoscopy, Affiliated Cancer Hospital and Institute of Guangzhou Medical University, Guangzhou 510091, China

## Abstract

Intestinal microbiota plays a key role in regulating the pathogenesis of human disease and maintaining health. Many diseases, mainly induced by bacteria, are on the rise due to the emergence of antibiotic-resistant strains. Intestinal microorganisms include organisms such as bacteria, viruses, and fungi. They play an important role in maintaining human health. Among these microorganisms, phages are the main members of intestinal viromes. In particular, the viral fraction, composed essentially of phages, affects homeostasis by exerting selective pressure on bacterial communities living in the intestinal tract. In recent years, with the widespread use and even abuse of antibacterial drugs, more and more drug-resistant bacteria have been found, and they show a trend of high drug resistance and multidrug resistance. Therefore, it has also become increasingly difficult to treat serious bacterial infections. Phages, a natural antibacterial agent with strong specificity and rapid proliferation, have come back to the field of vision of clinicians and scholars. In this study, the current state of research on intestinal phages was discussed, with an exploration of the impact of phage therapy against infectious diseases, as well as potential application beyond infectious diseases.

## 1. Background

Intestinal microorganisms are mainly composed of organisms such as bacteria, viruses, fungi, and protozoa. The interaction and homeostasis between various microorganisms are very important to human health. Bacteria and viruses are the two most abundant species in the intestinal microecosystem [1]. Phages are also the main members of intestinal viruses and play an important role in regulating intestinal microbiota. In recent years, with the widespread application of antibiotics, bacterial drug resistance has significantly increased globally. The treatment methods centered on the intestinal microecology have attracted widespread attention. Identifying the composition of intestinal phages and discovering their functions are not only a key point of microecology research but also a new entry point for protecting human health. The total number of bacteria in the human digestive tract exceeds 10^^14^. The intestinal microbiota of healthy people mainly composed of *Firmicutes*, *Bacteroides*, *Proteobacteria*, and *Actinobacteria*, accounting for over 90% of the total number of the intestinal bacteria [2]. Phages are the main components of intestinal viromes, containing up to 10^^8^ billion virus-like particles (VLPs) per mL of fecal filtrate [3]. According to their lytic ability, they are divided into lytic phages (virulent phages) and lysogenic phages (temperate phages). According to the difference in morphology, phages are divided into tailed phages, tailless phages, and filamentous phages and further divided into Siphoviridae, Myoviridae, Podoviridae, and so on. The main steps in the lysis of host bacteria by lytic phages can be summarized as follows: adsorption, invasion, replication, assembly, maturation, and lysis. After the phage is adsorbed on the cell surface of the host bacteria, the enzyme in the tail structure of the phage can penetrate the peptidoglycan layer of the host bacteria and the inner membrane, respectively, to release nucleic acid into the interior of the bacteria. The protein in the phage tail can also inhibit phage nucleic acid injected into the host bacteria from being excreted by them. When the integration of phage nucleic acid with the nucleic acid of host bacteria is completed, it will replicate with the bacterial nucleic acid and can be reassembled with the expressed protein shell to form a new progeny phage with bacteriolytic ability. Under the action of cytolytic enzymes and/or perforin, the infected bacteria are finally lysed, and the progeny phages released after lysis can continue to invade, infect, and lyse surrounding host bacteria in the same way. Since lytic phages can continuously proliferate and lyse bacteria after invading the host bacteria, resulting in the death of the host bacteria, virulent phages are generally used to treat bacterial infection. After the lysogenic phage integrates DNA or RNA into the nucleic acid of the host bacteria, the phage nucleic acid is continuously replicated with the replication of the host bacteria and constantly enters inside host bacteria cells of the next generation with the cell division of the host bacteria, which generally does not cause the lysis of the host bacteria. This phenomenon of simultaneous proliferation with the cellular host without lysing is called lysogenic conversion (lysogenic phenomenon). However, lysogenic conversion is not without lytic properties. Under certain external inducements, phage DNA or RNA integrated into the nucleic acid of the host bacteria can also spontaneously separate from the genomes of the host bacteria, enter the bacteriolytic cycle, and be assembled into a complete progeny phage, thus lysing the host bacteria.

Lysogenic phages are the main components in the human intestine. At present, studies have shown that the most widely distributed intestinal phages across the world are crAss-like phages [4], and healthy people in different regions are taken as the research object to have identified 23 core phages (phages shared by >50% of individuals) and 132 common phages (phages shared by 20∼50% of individuals) from them [5, 6]. Based on the characteristics of genome-wide average nucleotide identity (ANI), the International Committee on Taxonomy of Viruses (ICTVs) proposed a new virological classification system, in which sequence coverage exceeding 85% and ANI exceeding 95% are taken as thresholds, with comprehensive consideration of phylogenetic tree (PT) and gene-sharing networks (GSNs) for classification (Table1) [10]. The relationship between phages and human health can be discovered and their roles in disease diagnosis and treatment can be explored by the use of research into intestinal phageome.

## 2. The Main Mechanism of Action of Phages Affecting Body Health

### 2.1. The Regulation of the Composition of Intestinal Microbiota by Phages

#### 2.1.1. Predation

Phages can select target bacteria for “predation” by identifying specific membrane receptors on the bacterial surface. Generally speaking, bacterial infection by lytic phages first requires specific recognition via the structural proteins on the surface of lytic phages and adsorption of them to the surface receptors of host bacteria. This mainly depends on complementarity between protein in the phage tail and the molecular structure on the binding site of the bacterial surface [11]. Tailed phages hydrolyze peptidoglycan of cell walls mainly via lytic enzymes to release progeny phages. Lytic enzymes can lyse bacterial peptidoglycan by inhibiting peptidoglycan synthesis via a single protein or by enzymolysis of peptidoglycan via lysin and perforation-lysozyme systems [12]. There are mainly two kinds of enzymes that destroy bacterial biofilms: exolytic enzymes, which promote the entry of genomes into bacteria in the early stage, and endolytic enzymes, which degrade the host bacteria in the terminal stage to allow the release of progeny phages. After killing intestinal bacteria through this special “predation” effect, intestinal phages will also leave specific CRISPR spacer sequences on the bacterial genomes [13]. Through the identification of these sequences, it was found that bacterial death caused by natural phages in humans and animals is ubiquitous and plays an important role in the stability of intestinal microbiota. This kind of “predation” is mostly specific. For instance, *Faecalibacterium prausnitzii* phage can selectively infect *Faecalibacterium prausnitzii* and “turn a blind eye” to other intestinal bacteria [14]. However, macrogenomic analysis of the CRISPR spacer sequences also revealed that some intestinal phages have a wide range of bacterial hosts in human intestines [15].

#### 2.1.2. Lysogenic Conversion

Temperate phages compensate for their own adverse effects on host bacteria by improving the state of host bacteria, which in turn enhances adaptability and also endows the host with a new phenotype. This phenomenon is called “lysogenic conversion.” After entering the host bacteria, the temperate phage carries out gene integration, thereby avoiding recognition and clearance by macrophages and coexisting in the host bacteria for a long time [16]. Taylor et al. [17] and Wahl et al. [18] found that lysogenic conversion can increase the resistance of host bacteria to other phages, adhesion and colonization capability, environmental tolerance, and antibiotic resistance. The lysogenic conversion of phages not only improves the adaptability of phages to the intestine but also cooperates with host bacteria to enhance the evolutionary advantage.

#### 2.1.3. Seesaw Effect

Strains are exposed to the environment of antibacterial drugs and evolve into drug-resistant bacteria under genetic selection. The evolved strains lose the characteristics of phage resistance. Similarly, bacteria exposed to phage conditions lose their antimicrobial resistance after genetic selection [19]. For instance, Ho et al. [20] found that the mutation of bacterial gene EPAR leads to the reduction in adsorption of *Enterococcus faecalis* by phages; however, changes in this gene lead to an increased bacterial sensitivity of bacteria to daptomycin. When antibacterial drugs induce the phenotypic changes of bacteria, the ability of corresponding phages to prey on bacteria increases. This phenomenon of ebb and flow is also known as the “seesaw effect” [21].

#### 2.1.4. Epithelial Defense

Phages can reduce the colonization of pathogenic bacteria on the surface of the intestinal mucus layer, and they also phagocytize and lyse pathogenic bacteria [22, 23]. Barr et al. [24] found that in the human intestine, some phages adhere to the mucosa and reduce the diffusional movement of the phage itself, thus forming a structure similar to a defensive barrier with the epithelial tissue.

### 2.2. Phage-Mediated Immune Regulation

Another interesting feature of phages is their regulatory potential for an immune response [25]. Intestinal phages can actively scavenge invasive bacteria, reduce immune as well as inflammatory responses, and also maintain immune homeostasis [26]. Phage-mediated lysis is also involved in the generation of pathogen-associated molecular patterns (PAMPs), and PAMPs may translocate and activate immune response when intestinal permeability increases [27]. Phages can stimulate macrophages to phagocytize bacteria through opsonization to make them more easily enter the immune system [28]. The intestinal mucosa determines the interaction between phages and their hosts. Phage communities establish contacts with mucosal barriers to produce phage-mediated immune responses [29]. In this mode, innate immunity protects commensal microorganisms in the upper layer of mucus through lysis, and acquired immunity kills invasive pathogens in the deepest mucus through lysis [30]. In order to play an effective antibacterial role, adherent phages have to reduce bacterial colonization of mucus. Some phages express the proteins that display C-type lectin folds and immunoglobulin-type domains, interfering with the mucin expression of O-glycosylated MUC2 in the colon [31, 32]. On the other hand, pathogens that disrupt the innate immune response will be handled by the acquired immune system. Ig-type folds of phages were found in antibodies and T cell receptors [28]. Interestingly, the limiting factor of phages in the intestine is considered to be responsible for the production of specific immunoglobulin A (IgA). The results showed that if IgA levels are relatively low, phages will be found in feces; however, if IgA levels are elevated, there are no active phages in the feces, which may also directly explain the interaction between the relative abundance of intestinal phages with IgA-related immunity [28].

**Figure 1:**
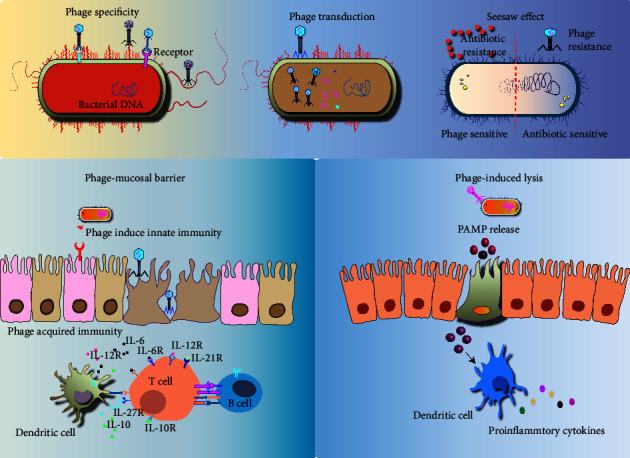
Mechanism of action of the phage therapy.

In addition, some phages can directly activate the intestinal immune pathway. Gogokhia et al. [31] found that phages can activate intestinal immunity via Toll-like receptor 9-dependent interferon signaling pathway and promote the proliferation of CD4+ and CD8+ T cells in Peyer's patch. Studies [33] also showed that *Escherichia coli* phages can play an immunosuppressive role and inhibit the expansion of intestinal immune cells, and adhesion proteins expressed by *Escherichia coli* phages can bind to lipopolysaccharide, which in turn control the lipopolysaccharide-mediated inflammatory response. Through the abovementioned important mechanisms of action, phages can reduce the invasion of foreign pathogens, increase the colonization ability of probiotics, and adjust the structure of intestinal microbiota, with the maintenance of the balance of intestinal microbiota, as well as the maintenance of intestinal homeostasis by regulation of intestinal immunity (Figure 1).

During the phage lytic infection cycle, phage-encoded binding proteins recognize and attach to receptors on the bacterial surface, such as fimbriae, flagella, porins, or receptor proteins on efflux pumps. The phage attaches and delivers the genomic content to the bacteria, viral replication occurs inside the cytoplasm; after assembly into new phage particles, the process repeats when the bacteria escape by lysing and then infect other susceptible bacteria. Phage therapy kills the target bacteria while at the same time strongly selecting for bacterial virulence or antibiotic resistance when the bacteria mutate to avoid a phage attack. In addition, phage therapy can mediate immune responses in the gut. The intestinal mucosa determines the interaction between phages and their hosts. Phage communities establish contacts with mucosal barriers to produce phage-mediated innate immune responses. Phage-mediated lysis is also involved in the generation of pathogen-associated molecular patterns (PAMPs), and PAMPs may translocate and activate immune response when intestinal permeability increases.

## 3. Relationship between Phages and Disease and Clinical Application of Phages

### 3.1. Phages and Infectious Diseases

At the beginning of the 20th century, phages preparations were successively successfully used in the treatment of bacillary dysentery, cholera, and so on [34]. Since then, more and more attempts have been made at phage therapy in various refractory infectious diseases. A research institute in Poland used phage therapy to treat 1307 patients with multidrug-resistant bacteria infections, and 85.9% of them were clinically improved or cured [35, 36]. Fecal filtrate prepared by extraction of phages from healthy human feces can effectively treat refractory *Clostridium difficile* infection [37]. Additionally, most of the phage preparations entering the clinical trial stage are also aimed at the infection of multidrug-resistant bacteria. The current phage preparations mainly focus on *Pseudomonas aeruginosa*, *Acinetobacter baumannii*, *Klebsiella pneumoniae,* and *Staphylococcus aureus* [38]. Dedrick et al. [39] reported a patient with cystic fibrosis caused by infection of *Pseudomonas aeruginosa* and *Mycobacterium abscessus*, whose symptoms significantly improved after 6 months of treatment with three-phage “cocktails.” Schooley et al. [40] reported a 68-year-old patient with diabetes mellitus infected with multidrug-resistant *Acinetobacter baumannii* (pancreatic pseudocyst), whose condition continued to worsen despite multiple antibiotics. Nine specific phages were screened out after analysis of the concentrated solution, and they were injected into the abscess cavity in combination with antibiotic therapy. The infection was effectively controlled after combined therapy. Bao et al. [41] reported that a 63-year-old patient with recurrent urinary tract infection caused by sulfamethoxazole-resistant *Klebsiella pneumoniae* recovered after treatment with phage “cocktail” therapy. Petrovic Fabijan et al. [42] treated 13 patients severely infected with *Staphylococcus aureus* combined with phage preparation AB-SA01, which not only effectively controlled the infection but also had no adverse reactions. The application of phage therapy in the treatment of infections at different sites is summarized as follows and shown in Table 2.

#### 3.1.1. Skin and Soft Tissue Infections

Infection is the main complication of severe burns, which may not only delay wound healing but also induce sepsis and even develop multiple organ dysfunction in severe cases. It is one of the important causes of burn-related death. *Pseudomonas aeruginosa*, *Klebsiella pneumoniae*, *Acinetobacter baumannii*, and *Staphylococcus aureus* are common bacteria with drug resistance. Phage therapy is also widely applied in the field of burns, and there are many cases with definite curative effects. Weber Dabrowska et al. [35] used phage “cocktail” therapy to treat burn patients by oral administration combined with topical application, and the pathogenic bacteria causing skin infection mainly cover different bacteria such as *Pseudomonas aeruginosa*, *Staphylococcus aureus*, *Klebsiella pneumoniae,* and *Proteus*. 85% (42/49) of these patients recovered, and bacteria were no longer detected in the topical skin. Although bacteria were still topically detected in the rest of the patients, the clinical symptoms improved significantly.

A chronic dystrophic ulcer is also prone to coinfections, which is common in diseases such as atherosclerosis and diabetes. Infection is an important influence factor for ulcer healing, and timely removal of infection can promote wound healing. Its pathogenic bacteria are dominated by *Staphylococcus aureus* and *Pseudomonas aeruginosa*, and their resistance to antibiotics often occurs, with the poor effect of long-term use of antibiotics [77]. Markoishvili et al. [43] applied a wound dressing impregnated with a variety of phages to treat patients with chronic ulcers, and the results showed that this dressing is not only effective but also safe. In a clinical trial of chronic venous leg ulcers related to bacterial infection conducted by Rhoads et al. [44], the “cocktail” phage WPP-201 was topically applied to leg ulcers. Although the wound healing rate of the phage-treated group was the same as that of the standard antibiotic treatment group, the adverse reactions of the former were significantly reduced compared with that of the latter. Morozova et al. [45] reported that patients with diabetic foot infections receive phage therapy. The previous antibiotic treatment was not effective for them. Their wounds were rinsed with phage preparations, covered with gauze soaked with phages agents for wet compress, with repeated replacement of gauze 4 times a day for 2-3 weeks, and their ulcers improved significantly. Fish et al. [46] successfully cured 6 patients with toe ulcers with commercial *Staphylococcus* phage SB-1. These patients responded poorly to conventional therapy, with no obvious adverse reaction after phage cure, and the ulcers did not recur. Kifelew et al. [47] found that the AB-SA01 phage cocktail can effectively treat diabetic wound infection caused by multidrug-resistant *Staphylococcus aureus* and also promote wound healing, whose efficacy is equivalent to vancomycin. Kumari et al. [48] found that in the mouse burn wound model, when the phage is applied topically, its protective efficacy is significantly higher than that of the antibiotics. Yin et al. [49] established a topical wound infection model in mice and confirmed that topical application of phages can effectively promote wound healing and has a better curative effect and higher safety than an injection of phages.

*Staphylococcus aureus* can not only cause skin and soft tissue infections but is also an important influence factor in many common skin diseases, such as chronic acne, atopic dermatitis, psoriasis, and eczema. Moreover, drug-resistant *Staphylococcus aureus* is one of the important factors for the protracted course and aggravation of skin diseases in these patients. Brown et al. [78] isolated 10 phages from the human skin and proved that these phages have lytic activity against acne. Totte et al. [50] reported that the patients with eczema, severe acne, and bacterial folliculitis for a long time were treated by local application of endolytic enzymes of phages (Staphefekt SA.100). The clinical symptoms of these patients improved after antibiotic treatment, but they were prone to relapse after drug withdrawal. After topical application of Staphefekt SA.100, *Staphylococcus aureus* was inhibited for a long time, the clinical symptoms of related skin diseases continued to improve, and the recurrence rate was significantly reduced. More importantly, Staphefekt SA. 100 is effective against both methicillin-susceptible bacteria and methicillin-resistant *Staphylococcus aureus* and also does not interfere with commensal skin microbiota or induce bacterial resistance [50].

#### 3.1.2. Oral Infection

Studies have shown that phages are one of the causes of oral health problems, especially related to the formation of dental plaque and the occurrence of periodontitis [79]. Naidu et al. [80] isolated phages from dental plaques of healthy people and proved the specificity and potential pathogenicity of phages among different individuals. Zhang et al. [81] proved that the virus and its host bacteria jointly promote the sustainable development of chronic periodontitis by genome analysis of phages isolated from patients with periodontitis. Ly et al. [82] found that there are differences in the composition of oral phages between healthy people and periodontitis patients: in the healthy population, long-tailed phages predominate; under the condition of periodontitis, the relative abundance of myotail phages in the subgingival tissue of patients significantly increases, whose changes may lead to the abnormal relative abundance of pathogenic bacteria in patients' plaques. Pride et al. [83] also found that lysogenic phages in the salivary virus community can store a variety of virulence factor genes, that phages may be a repository of pathogenic genes in the human oral cavity, and that phage imbalance may induce highly virulent pathogens, thus resulting in periodontal diseases. Tronstad et al. [84] found that phages can effectively prevent the formation of dental plaques. Machuca et al. [85] extracted phages of *Fusobacterium nucleatum* from healthy people, and *Fusobacterium nucleatum* is the pathogen of periodontitis and its excessive proliferation can mediate inflammation. Castillo-Ruiz et al. [51] extracted the phages of *Actinobacillus* from the flushing solution of tooth cleaning for the treatment of periodontitis. In recent years, genetically engineered phages have also played an important role in oral diseases. Guo et al. [52] found that the specifically targeted antibacterial peptides produced by genetically engineered phages can efficiently and selectively kill the cariogenic pathogen *Streptococcus mutans*, thereby reducing the occurrence of dental caries. Smith [86] successfully inserted the genetic information at antibody-antigen binding sites into phage DNA and screened out the phages that can bind highly to antibody specificity, which provided new inspiration for improving the targeting of drugs. Both periodontitis and root canal infection are closely related to *Enterococcus faecalis* infection. Some phages have been found to specifically attack *Enterococcus faecalis*, such as phages EF24C [44] and EFDG1 [87]. Tinoco et al. [53] studied the response of phage *ϕ*Ef11 after its infection with *Enterococcus faecalis*. They found that phages can convert prophages into lytic phages by deleting the phage repressor genes and evade the host's inhibitory response by replacing the initiation factor, and the modified phage can effectively destroy bacterial biofilms, thereby improving periodontitis and root canal infection. Xu et al. [54] and Li et al. [55] proved that phage lytic enzyme ClyR can selectively kill bacteria that can cause dental caries and *Enterococcus faecalis* that can induce periapical dental pulp infection, but it is harmless to common oral commensal bacteria. Also, it has been confirmed by rat dental caries model experiments that phage lytic enzyme ClyR can significantly reduce the degree of dental caries and has a potential therapeutic effect on periodontal disease.

#### 3.1.3. Gastrointestinal Infections

The common pathogenic bacteria of gastrointestinal infections include *Escherichia coli*, *Campylobacter jejuni*, and *Salmonella shigella*, . Some uncommon pathogenic bacteria such as *Vibrio cholerae* and *Clostridium difficile* can also cause acute and chronic infectious enteritis. Phage preparation was first successfully used in the treatment of bacillary dysentery and cholera [34]. Subsequently, Ott et al. [37] prepared fecal sterile filtrate from healthy human feces, which can effectively treat refractory *Clostridium difficile* infection, and it is speculated that the phages in the filtrate play a therapeutic role. Bruttin and Brussow [56] and Sarker et al. [57, 88] used *Escherichia coli*-T4 phage to treat acute bacterial infectious diarrhea in adults and children. They found that although *Escherichia coli*-T4 phage is safe, there is still controversy about its therapeutic efficacy. Enteropathogenic *Escherichia coli* (EPEC) is a drug-resistant *Escherichia coli* causing severe diarrhea. In recent years, the resistance of EPEC to commonly used antibiotics has been increasing. Vahedi et al. [58] found by comparing the therapeutic effects of phages and ciprofloxacin in the mouse model infected with EPEC that phage therapy can not only reduce the content of EPEC in vivo but also ensure the normal growth of mice. Jaiswal et al. [59] employed the model of rabbit infected with *Vibrio cholerae*. They found that phage “cocktail” treatment given 6 hours after infection with *Vibrio cholerae* in rabbits can significantly reduce the proliferation of *Vibrio cholerae*, but this efficacy of phages may be related to the administration time. If the drug is administered within 6 hours of infection, the number of *Vibrio cholerae* cannot be reduced. Nale et al. [60] confirmed through the hamster model that phage “cocktail” therapy can significantly reduce the colonization of *Clostridium difficile*. In addition, Galtier et al. [61] demonstrated that phages can effectively reduce intestinal pathogens, and phages have less impact on the composition of intestinal microbiota than antibiotics.

#### 3.1.4. Respiratory Infection

The respiratory tract is the cavity through which the human body communicates with the outside world, and bacterial infection is also a common cause of respiratory diseases. Similarly, phage therapy also shows a good curative effect on respiratory infection. In the study by Cao et al. [62], multidrug-resistant *Klebsiella pneumoniae* was inoculated into the lungs of mice, and then, the mice could be protected from fatal pneumonia by nasal inhalation of *Klebsiella pneumoniae* phage. The research team also used phages to treat a mink model of lung infection, and the result showed that the survival rate of minks after phage treatment is 80%. Alemayehu et al. [63] found that after the lungs of mice are infected with *Pseudomonas aeruginosa*, the bacterial load in the lungs of mice can be reduced to an undetectable level after nasal inhalation of phage preparations, while *Pseudomonas aeruginosa* proliferates in large quantities in the lungs of the mice not treated with phage preparation. Oduor et al. [64] found that an intravenous injection of phage preparation can treat *Staphylococcus aureus*-induced pneumonia in mice and significantly inhibit the bacterial load (from 8 CFU/g to 0.5 CFU/g). In a study of chronic lung infection, Waters et al. [65] found that after the lungs of mice are infected with *Pseudomonas aeruginosa*, nasal inhalation of phage can effectively reduce the number of bacteria in the lungs of mice, which also suggests the effectiveness of phage therapy in the treatment of chronic respiratory infection.

#### 3.1.5. Urinary Tract Infection

Urinary tract infections are mostly anaerobic bacterial infections in the intestine. Patients with more underlying diseases, critical illnesses, or long-term indwelling urinary catheters are prone to complicated/refractory bacterial infections. Bao et al. [41] reported that a 63-year-old female patient with recurrent urinary tract infections secondary to extensively drug-resistant *Klebsiella pneumoniae* infection was successfully cured after two rounds of phage “cocktail” therapy combined with antibiotics. Patients with long-term indwelling catheters are prone to urinary tract infection. Garretto et al. reported [89] that after phages are smeared against *Pseudomonas aeruginosa*, *Escherichia coli,* and *Proteus mirabilis* on the indwelling catheter, bacterial biofilm formation on the catheter can be significantly reduced, thus playing an anti-infection role. Leitner et al. [66] conducted a randomized, double-blind, prospective clinical study of intravesical Pyo-phage in patients with postprostatectomy infection. In this study, patients with positive urine culture were selected, the sensitivity of isolated pathogens to phage/antibiotics was first determined, and the subjects with sensitive test results were randomly divided into three groups (phage therapy, antibiotic group, and placebo group); after one week of phage/antibiotic/placebo treatment, it was found that the phage group is better than the antibiotic group, and the effective rate is 66.67% (bacterial titers decrease significantly), with no adverse reactions. In addition, Kuipers et al. [67] and Rostkowska et al. [68] both reported the successful treatment of refractory urinary tract infection with *Klebsiella pneumoniae* with phages in renal transplant patients after surgery.

#### 3.1.6. Eye Infection

Fukuda et al. [69] topically applied eye drops containing *Pseudomonas aeruginosa* phages to the cornea of normal mice. The result showed that the eye drops do not cause inflammatory reactions such as corneal opacity or inflammatory cell infiltration. Then, a mouse model of *Pseudomonas aeruginosa*-induced keratitis was selected, followed by administration of phage-containing eye drops after infection. The result showed that after 5 days, the corneal structure of the mice in the experimental group basically returned to normal, while most of the corneas of the mice in the control group without phage treatment are perforated. Furusawa et al. [70] also administrated a mouse model of corneal infection with *Pseudomonas aeruginosa*; after 30 minutes and 1, 3, 6, and 12 hours of corneal infection with *Pseudomonas aeruginosa* in mice, respectively, eye drops containing two kinds of phages were administrated. The results showed that with the administration of phage eye drops within 3 h after corneal infection, the bacterial count of mouse cornea is significantly decreased compared with that in the control group, indicating that phage eye drops have an antibacterial effect.

#### 3.1.7. Ear Infection

Wright et al. [71] conducted a clinical trial evaluating the phage preparation in the treatment of chronic otitis media, 24 patients with chronic otitis media caused by drug-resistant *Pseudomonas aeruginosa* infection lasting for several years were randomly divided into two groups, and they were treated with phages and placebo, respectively. The results showed that the number of *Pseudomonas aeruginosa* in the phage treatment group significantly decreases, and no local or systemic side effects are found, while the bacterial count in the placebo group does not change significantly. In an animal experiment administrated by Hawkins et al. [72], a cocktail preparation containing 6 different phages was injected into the ear canal of dogs with chronic otitis media caused by *Pseudomonas aeruginosa* infection, and the clinical score and bacterial count were found to decrease after 48 h, without the identification of no adverse reactions.

#### 3.1.8. Nasal Infection

Ooi et al. [73] conducted a phase 1b clinical study. Nine patients with chronic sinusitis were treated with AB-SAO1 (a mixture of three natural *Staphylococcus aureus* phages). The results showed that intranasal phage therapy is safe and well tolerated and has a significant effect on chronic sinusitis caused by *Staphylococcus aureus*.

#### 3.1.9. Sepsis/Bacteremia

Sepsis refers to the acute systemic infection caused by various pathogenic bacteria invading the blood circulation, growing, multiplying, and producing toxins in blood. If the bacteria invading the bloodstream are removed by the human body's defense function and there are no obvious symptoms of toxemia, it will be called bacteremia. Schneider et al. [74] injected a lethal dose of *Escherichia coli* into mice, resulting in bacteremia, followed by administration of the same dose of phage 10 min and 60 min after bacterial injection, respectively. It was observed that the survival rates of mice are 100% and 95%, respectively, indicating that the administration of phages within a short time after infection can effectively improve the survival rate, but no mice survive when phages are administrated 3 h after bacterial infection. Another study by Pouillot et al. [75] showed that in a rat model of bacteremia caused by *Escherichia coli*, the survival rates of mice are 100% and 50% when phages are administrated 7 h and 24 h after bacterial injection, respectively. In addition to mouse models, Jang et al. [90] used drosophila models to evaluate the efficacy of phages in the treatment of bacteremia. The establishment of the bacteremia model is by injection of a lethal dose of *Pseudomonas aeruginosa* into *Drosophila melanogaster*, and the model group was fed with phage. The results showed that phages can significantly delay or prevent the death of *Drosophila melanogaster*.

#### 3.1.10. Novel Coronavirus Pneumonia

Phages are easier to induce the body to produce an immune response due to their immunogenicity and also have advantages such as stability and low cost, and thus, they have been widely studied as a vaccine carrier [91]. Phage display technology (PDT) is to insert the gene sequence of encoding exogenous polypeptide or protein into the coat protein gene of phages to display them on the surface of phage coat protein in the form of the fusion protein, and also, they can maintain good biological activity and original biological function, thereby facilitating the identification and combination of target molecules. This technology has been widely applied in many fields such as immunology, oncology, and pharmacology, with the important value in pathogen detection, disease treatment, vaccine, or drug design [92]. Therefore, the comprehensive use of phage immunogenicity and PDT have a good application prospect in the development of a novel coronavirus vaccine. Staquicini et al. [93] reported the development and research of a novel coronavirus-targeted vaccine based on phages. After analyzing the structure of protein S, the researchers selected 6 antigen epitopes and cloned them into phage vector genomes, respectively. Epitope 4 showed that phages can induce sustained specific humoral immunity in mice without other adverse reactions. After that, the researchers also screened the peptide that can bind to the surface of the pulmonary airway and alveolar inner wall cells through the random peptide library of phages, cloned, and inserted it into phage to construct phage particles. Additionally, they also found that phage particles can induce stronger immune responses [94]. Li et al. [76] identified a phage Ab8 that can bind to antibody V with high affinity by establishing a human antibody V domain phage-displayed library. Through further experiments, they found that the phage can neutralize severe acute respiratory syndrome coronavirus 2 (SARS-CoV-2) in wild-type mice and shows good preventive and therapeutic effects in a hamster model of SARS-CoV-2 infection.

### 3.2. Phages and Noninfectious Diseases

**Figure 2:**
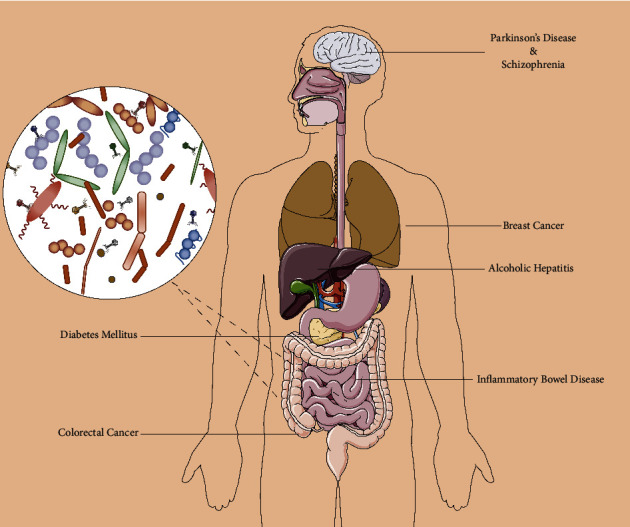
Phages for diagnosis and treatment of diseases beyond infection.

Numerous studies have shown that there are significant differences in the diversity and structure of intestinal phageomes between healthy people and patients (especially patients with chronic diseases) [95–97]. Therefore, in-depth analysis of intestinal phageomes can provide new ideas for revealing the pathological mechanism of various diseases and help find new diagnostic and therapeutic markers. As shown in Figure 2, intestinal phages play an important role in the development of inflammatory bowel disease, alcoholic liver disease, diabetes, colorectal cancer, breast cancer, Parkinson's disease, and schizophrenia.

With the application of viromics, the role of phages as biomarkers in disease diagnosis has been gradually discovered. Phage therapy as a targeted bactericidal method plays a considerable role in the treatment of many diseases beyond infection.

#### 3.2.1. Inflammatory Bowel Disease

Inflammatory bowel disease is a chronic intestinal inflammatory disease caused by the interaction between multifactors such as genetics, immunity, microbes, and environmental factors, mainly including ulcerative colitis and Crohn's disease [98]. The long-term recurrent digestive symptoms and other system dysfunction have placed a heavy burden on patients. Studies [6] have confirmed that there are significant differences in the diversity and structure of intestinal viruses between patients with inflammatory bowel disease and healthy members of their families, and they are related to the progression of inflammatory bowel disease. The decreased relative abundance of *Faecalibacterium prausnitzii* in the intestine of patients with inflammatory bowel disease is an important microbiota feature of inflammatory bowel disease. Further analysis showed that the abundance of a certain phage is related to that of *Faecalibacterium prausnitzii*, and it is speculated that the increased mortality of *Faecalibacterium prausnitzii* in the intestine of patients with inflammatory bowel disease is mediated by this phage [14]. Zuo et al. [99] conducted a metagenomic analysis of the samples of domestic patients with ulcerative colitis and found that there is a significant increase in intestinal tailed phages, accompanied by a significant increase in the abundance of *Escherichia* phages and *Enterobacteria* phages. Similarly, the inflammatory microenvironment in the intestine may increase the induction of prophages, promote lysis of certain bacterial species, and aggravate microbiota imbalance and intestinal inflammation. The study of Yang et al. [100] suggested that intestinal phages in healthy people can inhibit the occurrence of intestinal inflammation by producing interferon-*β* mediated by Toll-like receptor 3 and Toll-like receptor 7. Van belleghem et al. [101] also observed that phages can directly interact with innate immune cells, and when peripheral blood monocytes are incubated in vitro, *Staphylococcus aureus* phages or *Pseudomonas aeruginosa* phages can induce the transcriptional response of monocytes and significantly increase the transcription of interleukin-1*β*, interleukin 6, and tumor necrosis factor-*α*, which in turn aggravate intestinal inflammation.

#### 3.2.2. Alcoholic Hepatitis

Alcoholic hepatitis is a disease associated with a high intake of alcohol. Studies have found that *Enterococcus faecalis*, an intestinal bacterium, may be involved in the occurrence of alcoholic hepatitis [102]. Duan et al. [103] analyzed the fecal samples from patients with alcoholic hepatitis and healthy people. They found that the level of *Enterococcus faecalis* in the fecal samples from patients with alcoholic hepatitis is 300 times higher than that of healthy people, and the relative abundance of *Enterococcus faecalis* significantly increases in the fecal samples of about 80% of alcoholic hepatitis patients. Through further analysis, they found that there is a gene that can encode cytolysins in about 30% of *Enterococcus faecalis*. The mouse model of alcoholic hepatitis was established by the use of a high alcohol diet. After fecal transplantation containing cytolysin, these alcoholic hepatitis mice show a certain degree of hepatocyte damage and even death, while the mice with alcoholic hepatitis transplanted with cytolysin-deficient fecal samples do not show liver damage. The phage of *Enterococcus faecalis* can reduce the abundance of cytolysin-containing *Enterococcus faecalis* and alleviate liver damage in mice with alcoholic hepatitis by targeting, thus playing a protective role.

#### 3.2.3. Diabetes Mellitus

Diabetes mellitus is a group of clinical syndromes characterized by chronic hyperglycemia, with the characteristics such as many complications, hard-to-cure, slow onset, and protracted course. It can cause complications in organs such as kidneys, nerves, and blood vessels, which not only affect the quality of life in patients but also become a public health problem that seriously threatens human health. Ma et al. [104] found that the number of intestinal phages in type 2 diabetes patients is significantly higher than that in healthy individuals, and seven phages are closely related to the occurrence and development of type 2 diabetes. Moreover, the changes in intestinal phages in type 2 diabetes patients are related to changes in the relative abundance and diversity of host bacteria. Zhao et al. [105] analyzed the intestinal microbiota and viral genomes of children at high risk of type 1 diabetes and healthy children. They obtained the following findings: viral genomes of children at high risk for type 1 diabetes change before the onset of disease, and the differences between the two groups increase with age; phage genome sequences are closely related to type 1 diabetes, and before the change in serological parameters of patients with type 1 diabetes, the diversity and relative abundance of intestinal phages significantly decrease, while the intestinal microbiota does not significantly change. This suggested that the change of intestinal microbiota during the development of autoimmune diseases in patients with type 1 diabetes may be caused by the change of phages.

#### 3.2.4. Colorectal Cancer

Colorectal cancer is one of the most malignant tumors with extremely high morbidity and mortality. There are no obvious symptoms in the early stage, and most patients are diagnosed in the middle and late stages. In many studies, it has been reported that the microbiota disturbance in colorectal cancer patients is related to the occurrence and development of colorectal cancer [106]. The diversity of intestinal phage community in colorectal cancer patients significantly increases compared with that of healthy people, and it has been also found that dysregulation of intestinal viromes is related to the progression of colorectal cancer, and there are more than 20 virus genera that differ significantly between colorectal cancer patients and healthy people [107]. Hannigan et al. [108] identified the viruses with significantly altered composition in fecal samples of colorectal cancer patients, mainly the phages from Siphoviridae and Myoviridae. Zheng et al. [109] obtained the following findings: *Fusobacterium nucleatum* increases in the feces of colorectal cancer patients, while butyrate-producing bacteria such as *Clostridium butyricum* decrease; *Fusobacterium nucleatum* is enriched in tumors and can induce autophagy of colorectal cancer cells through Toll-like receptor 4-myeloid differentiation factor 88 pathway to resist chemotherapy, while the butyric acid secreted by *Clostridium butyricum* can inhibit colorectal cancer cells and further isolate *Fusobacterium nucleatum* phages from saliva, which can be linked to nanoparticles loaded with colorectal cancer chemotherapy drugs; in animal experiments, the drug was found to be enriched in colorectal tumors of APC^min/+^ mice; this significantly prolongs the survival time of colorectal cancer mice, reduces the occurrence of intestinal adenoma and the abundance of *Fusobacterium nucleatum*, and increases the levels of *Clostridium butyricum* and colonic butyric acid.

#### 3.2.5. Breast Cancer

Breast cancer often metastasizes to the bone, lung, liver, brain, and so on and causes serious secondary diseases [110]. Currently, new treatment methods for breast cancer mainly include new technologies such as oncolytic virus therapy and virus and phage display immunotherapies. Phage display immunotherapy presents polypeptide or protein antigen by gene fusion with phage coat protein, and phage structure preparation can be used as protective or preventive vaccines against cancer [111]. In addition, filamentous phages can be used as a carrier to combine with anticancer drugs to deliver drugs to cancer cells for targeted therapy [112].

#### 3.2.6. Parkinson's Disease

Parkinson's disease is a common neurodegenerative disease in the elderly, which is related to factors such as heredity, environment, and nervous system aging. Tetz et al. [113] analyzed fecal samples from 32 Parkinson's disease patients and 28 healthy people. After the comparative analysis of bacterial and phage community composition, it was found that *Lactobacillus*, *Streptococcus,* and *Lactococcus* significantly decrease in Parkinson's disease patients. In healthy people, the number of virulent and temperate *Lactococcus* phages is roughly the same, while virulent phages dominate in Parkinson's disease patients. The increase of lytic c2-like *Lactococcus* phages and 936-like *Lactococcus* phages may have a scavenging effect on intestinal *Lactococcus*, and *Lactococcus* plays an important regulatory role in the production of dopamine. Therefore, the reduction in phage-mediated *Lactococcus* may accelerate the development of Parkinson's disease.

#### 3.2.7. Schizophrenia

Schizophrenia is a mental disorder with a complex mechanism. In recent years, with the in-depth study of the brain-gut axis, more and more psychiatric diseases have been found to be related to intestinal microecology. Yolken et al. [114] recruited 41 schizophrenic patients and 33 healthy people and collected DNA samples with throat swabs for metagenomic analysis. Studies showed that *Lactobacillus phage phiadh* significantly increases in schizophrenic patients. Of the 41 schizophrenic patients included in the study, the sequencing results of 17 patients matched *Lactobacillus phage phiadh* in one or more places, while there was only one positive match in the healthy control group. After further analysis of drug use in patients, it was found that the abundance of *Lactobacillus phage phiadh* is also negatively correlated with the use of sodium valproate. 17 patients who did not use sodium valproate tested positive for *Lactobacillus phage phiadh*.

## 4. PDT Clinical Application

PDT is a molecular biology technology that uses phage as a carrier to display foreign proteins on the phage surface in the form of fusion expression by integrating exogenous polypeptide genes into phage ones. The PDT clinical application mainly focuses on the following aspects. (1) In antibody production, high-specific monoclonal antibody genotypes can be screened quickly and efficiently through PDT, and corresponding monoclonal antibodies can be obtained after large-scale expression in a prokaryotic system, with the advantages of fast speed and no need for immune animals. In 1993, the human anti-TNF-*α* antibody D2E7 was produced by PDT [115]. In 2002, D2E7 was officially renamed as adalimumab and FDA-approved for the clinical treatment of rheumatoid arthritis and then approved for the treatment of psoriasis and inflammatory bowel disease. Afterwards, more and more human antibodies were applied in the clinical practice, including anthrax infection—raxibacumab, systemic lupus erythematosus—belimumab, nonsmall-cell lung cancer—necitumumab, gastric, and colorectal cancer—ramucirumab, and so on [116]. (2) In vaccine development, phage-displayed vaccines can display exogenous antigens on the surface of the phage. With the phage proliferation, the expression level of exogenous antigens is also increased so that the body can produce a high immune reaction. Especially in major life-threatening infectious diseases, PDT plays an important role. In addition to the aforementioned COVID-19, Khurana et al. [117] used PDT to develop Ebola virus vaccines; Li et al. [118] adopted PDT to screen single-chain variable fragments targeting influenza virus hemagglutinin subunit 2 (HA2) for influenza vaccine development. (3) In disease diagnosis and treatment, PDT can be utilized to target and identify tumors and organs. The polypeptide can directly target tumor cells or tissues after being coupled with cancer therapeutic drugs by screening the polypeptides specifically bound to tumor cell antigen epitopes. And if fluorescent groups are bound to these specific binding polypeptides, they can also be used for early cancer screening and detection of tumor lesion sites [119]. For example, Shen et al. [120] identified potential ligands of prostate-specific membrane antigen (PSMA) suitable for further development as novel PSMA-targeted peptides by PDT, and the two of the peptide sequences deduced from DNA sequencing of binding phage were SHSFSVGSGDHSPFT and GRFLTGGTGRLLRIS, which may be the basis for further development of peptides for prostate cancer tumor imaging and therapy. Hou et al. [121] adopted PDT to select novel binding peptides for early colon cancer imaging detection and found a peptide termed CBP-DWS, which was demonstrated to be capable of binding to a panel of human colon cancer cell lines and tissues by targeting glypican-3. Han et al. [122] reported a novel 12-mer peptide, GP-5 (IHKDKNAPSLVP), binding to gastric cancer cells specifically and sensitively, providing support for the speculation that the peptide GP-5 is a potential candidate to be developed as a useful molecule fragment for the imaging detection and targeted therapy of gastric cancer.

## 5. Discussion

As phage therapy has attracted the attention of clinicians and scholars again, its advantages and disadvantages are also known. The advantages of phage therapy are as follows: first, phage specificity is high. General antibiotics are relatively broad-spectrum and the elimination of target pathogens by them also causes destruction of other symbiotic microbiota. Thus, long-term use of antibiotics is not only easy to cause microbiota disturbance but may also lead to opportunistic infection. However, phages can specifically infect host bacteria without affecting other symbiotic microbiota; second, phages have low resistance to bacteria. With the use of antibiotics, the incidence of bacterial resistance to drugs is increasing, while the incidence of bacterial resistance to phages is far below that of antibiotic resistance, and phages can “co-evolve” with bacteria to reduce the incidence of drug resistance [123]; third, the phages, which has the strong bacteriolytic ability, can destroy the bacterial biofilm structure by secreting enzymes such as hydrolase and polysaccharide depolymerases, while antibacterial drugs have limited efficacy against bacterial biofilms [124]; and finally, as a natural antibacterial substance, phages can self-replicate, with a short proliferation cycle. The R&D cycle of phages is shorter than that of general antibacterial drugs. However, phage therapy also has the following common limitations: first, the antibacterial spectrum of phages is narrow. Due to the specific recognition of host bacteria by phages, their curative effect can be significantly reduced in the treatment of multiple infections. Therefore, it is usually necessary to employ the “cocktail therapy” containing multiple phages, which also increases the production cost [125]; second, phages have low resistance to bacterial CRISPR systems. The bacterial CRISPR system can lyse phages and DNA to resist phages. At present, there is no effective way to avoid this defense system [126]; and third, there are also potential risks for phage therapy, such as the potential transfer of virulence or antibiotic resistance genes to infected bacteria, which may lead to such risks as the emergence of highly pathogenic strains [127].

Antibacterial Resistance Leadership Group (ARLG) made recommendations on the potential clinical conditions, laboratory tests, and pharmacokinetics that may need to be considered in phage therapy. Among them, the administration route, dose, frequency, and duration all have indicated that the optimal method has not yet been established. There are currently insufficient data to support a definitive recommendation on the optimal frequency and duration of phage therapy for any given route of administration or a specific type of infection. Extant data suggest that phage needs to be administrated repeatedly to maximize the phage concentration at the infected site, but the ideal frequency and duration of administration are still unclear. The dose and duration required may vary with factors such as phage products, pathogens, disease burden, and infection location [128].

Another aspect that cannot be ignored is that the limitations of phage detection technology also affect our use of similar 16S sequencing methods to make a large-sample analysis of population samples and in turn summarize the phage characteristics of different diseases, which also brings inconvenience to the formulation of phage treatment plans. Temperate phages account for 20–50% of the free phages in the human intestine, and their proportion differs among studies depending on sample sizes and detection methods. For instance, in a study of comparing the virome content of twins, the proportion of temperate phages was estimated according to the copy number of integrase genes, which encodes the enzyme integrating temperate phage genes into bacterial chromosomes [129]. However, since many temperate phages exist as self-replicating free bodies in the host cells and therefore do not express integrase, their copy number is only a rough estimate of the lower limit of the proportion of temperate phages. In addition, the distribution of prophages in the intestinal microbiota is difficult to gauge since most bacterial strains express lysozyme (usually multiple lysozymes), especially *Bacteroides*, *Firmicutes*, *Actinobacteria*, and *Proteobacteria* [130]. Although metagenomic sequencing has confirmed the presence of prophages in the intestinal microbiota [131], it cannot distinguish between active and defective prophages. Since the latter do not undergo the recovery and dissolution cycle due to mutation, most phages detected by genome sequencing are active phages [132, 133]. Edwards et al. [134] predicted the hosts of different phages by three methods: (1) nucleotide similarity search between phages and bacterial genome, (2) BLAST search, and (3) longest exact nucleotide match between phages and bacterial genomes. However, the predictive accuracies for 820 intact phages are only 37% and 40%. Although CRISPR spacer sequencing can significantly improve the accuracy and efficiency of phage host prediction [15], it can only identify hosts that encode the CRISPR-Cas system. Furthermore, phage infection in these hosts is relatively late, and only 4–13% of the phages can find the target host. Large datasets by Paez Espino et al. [135] and WiSH are used for predicting the hosts of 59% overlapping groups [136]. In a recent study [137], 180 persistent phage clusters were identified by a combination of both methods and 1/3 of which may be related to some bacterial genera (*Faecalibacterium* and *Bacteroides*). However, the predictive accuracy and efficiency of either single or combined methods are fuzzy.

In general, the growing problem of antibiotic resistance has brought phages back to the field of vision of clinicians and researchers. In recent years, there have been more and more research studies on phage therapy. According to the clinical trials, there have been 41 clinical studies of phages/phage lytic enzyme therapy (Table 3). From single/cocktail phage therapy, phage lytic enzyme therapy to fecal phage filtrate transplantation, phage therapy is also changing dramatically in form. The advantages of phages, such as high host specificity and low resistance, undoubtedly provide an important breakthrough direction for solving the dilemma of bacterial drug resistance, but phages also have limitations such as potential virulent risks and a narrow antibacterial spectrum. Therefore, the current research is still focused on clinical cases and animal experiments. In these studies, although phage therapy has shown good efficacy and safety, many problems still need to be overcome before it can be officially put into clinical application.

## Figures and Tables

**Table 1:** Human intestinal phage classification.

Reference	Year	Location	Sample size	Object	Phage	Host	Identification method
Dutilh et al. [4]	2014	United States	12	Humans (twins)	crAssphage	*Bacteroides*	PT

Devoto et al. [7]	2019	United States	212	Humans from Bangladesh and Tanzania, two African baboon social groups, and Danish pigs	Lak phage	*Prevotella*	PT

Camarillo-Guerrero et al. [8]	2021	United Kingdom	28060	Humans	Gubaphage	*Bacteroides* and *Parabacteroides*	PT

Benler et al. [9]	2021	United States	5742	Humans	Quimbyviridae	*Prevotella*, *Bacteroides*, and *Parabacteroides*	PT

Benler et al. [9]	2021	United States	5742	Humans	Flandersviridae/Gratiaviridae	*Bacteroides* and *Parabacteroides*	PT and GSN

PT, phylogenetic tree; GSN, gene-sharing networks.

**Table 2:** Phage therapy in different infectious diseases.

Disease type	Reference	Year	Location	Disease	Object (*n*)	Study type	Host	Outcome
Skin and soft tissue infections	Weber Dabrowska et al. [35]	2000	Poland	Pyogenic infections of burns	Human (49)	Single arm study	*Staphylococcus aureus*, *Escherichia coli*, *Klebsiella*, *Proteus*, and *Pseudomonas*	86% full recovery while 14% marked improvement.
	Markoishvili et al. [43]	2002	United States	Poorly vascularized and venous stasis ulcers	Human (96)	Single arm study	*Pseudomonas aeruginosa*, *Escherichia coli*, *Staphylococcus aureus*, *Streptococcus*, and *Proteus*	The wounds/ulcers healed completely in 67 (70%) out of 96 patients. In 22 cases in which microbiologic data were available, healing was associated with the concomitant elimination of, or a reduction in, specific pathogenic bacteria in the ulcers.
	Rhoads et al. [44]	2009	United States	Venous leg ulcers	Human (42)	RCT	*Pseudomonas aeruginosa*, *Staphylococcus aureus*, and *Escherichia coli*	No adverse events. No significant difference of healing compared to antibiotic control.
	Morozova et al. [45]	2018	Russian	Infected diabetic foot ulcers	Human (2)	Case report	*Staphylococcus*, *Enterococcus*, and *Pseudomonas aeruginosa*	Wound continues to improve, while MRSA infection is not detected.
	Fish et al. [46]	2018	United States	Infected diabetic toe ulcers	Human (6)	Case report	*Staphylococcus aureus*	No adverse effects, tissue breakdown, or recurrence of infection were seen
	Kifelew et al. [47]	2020	Australia	Diabetic wound infections	Balb/c mice (48)	Animal experiment	*Staphylococcus aureus*	In phage-treated mice, wound healing was seen as similar to vancomycin treatment. No mortality was recorded associated with infections, and postmortem examinations did not show any evident pathological lesions other than the skin wounds. No adverse effects related to the application of phages were observed.
	Kumari et al. [48]	2011	India	Burn wound infection	Balb/c mice (30)	Animal experiment	*Klebsiella pneumoniae*	Significant reduction in mortality and more effective than silver nitrate and gentamicin.
	Yin et al. [49]	2017	China	Wound infection	Balb/c mice (36)	Animal experiment	*Acinetobacter baumannii*	Wound sizes in animals receiving locally applied phage were significantly smaller, drier, and cleaner than in mice receiving either systemically administered phage or no treatment. Infected mice receiving no treatment succumbed rapidly. In contrast, all mice treated with phage or polymyxin B survived the entire 7 days of the observation period.
	Totte et al. [50]	2017	Netherlands	Acne vulgaris and eczema	Human (3)	Case report	*Staphylococcus aureus*	Reduction and prevention of clinical symptoms and does not interfere with the commensal skin microbes and is also not expected to induce bacterial resistance.

Oral infection	Castillo-Ruiz et al. [51]	2011	Chile	Periodontitis	17 clinical samples were obtained from saliva and wastewater from dental chair drainages (NA)	In vitro	*Aggregatibacter actinomycetemcomitans*	Kill 99% of the bacteria within a biofilm.
	Guo et al. [52]	2015	United States	Dental caries	20 bacterial species, including multiple oral *Streptococcus* (NA)	In vitro	*Streptococcus*	Potent in killing *Streptococcus mutans* and *Streptococcus salivarius*.
	Tinoco et al. [53]	2017	Brazil	Dentin infection	*Enterococcus faecalis V583* (vancomycin resistant strain) or *Enterococcus faecalis JH2-2* (fusidic acid and rifampin resistant, vancomycin sensitive strain) (NA)	In vitro	*Enterococcus faecalis*	The recovered *Enterococcus faecalis* titer was reduced by 18% for the *Enterococcus faecalis JH2-2* infected models and by 99% for the *Enterococcus faecalis V583* infected models.
	Xu et al. [54]	2018	China	Dental caries	Sprague Dawley rats (36)	Animal experiment	*Streptococcus*	*Streptococcus mutans* and *Streptococcus sobrinus* biofilms are significantly decreased after treatment with ClyR for 5 min. Furthermore, continuous administration of ClyR for 40 days significantly reduced the severity of caries in rat models infected with a single or a mixed bacteria of *Streptococcus mutans* and *Streptococcus sobrinus*.
	Li et al. [55]	2018	China	Endodontic infection	Caries-free single-rooted teeth selected from orthodontic extraction (NA)	Ex vivo dental model	*Enterococcus faecalis*	ClyR degrades *Enterococcus faecalis* biofilm with high efficacy in a dose-dependent manner.

Gastrointestinal infections	Ott et al. [37]	2016	Germany	Diarrhea	Human (5)	Case report	*Clostridium difficile*	Sufficient to restore normal stool habits and eliminate symptoms.
	Bruttin and Brussow [56]	2005	Switzerland	Healthy volunteers to measure the bioavailability of oral phage for diarrheal diseases	Human (15)	Single arm study	*Escherichia coli*	Safe
	Sarker et al. [57]	2016	Bangladesh	Diarrhea	Human (120)	RCT	*Escherichia coli*	No adverse events. Fecal coliphage was increased in treated over control children, but the titers did not show substantial intestinal phage replication but no amelioration in quantitative diarrhea parameter by phage therapy.
	Vahedi et al. [58]	2018	Iran	Diarrhea	Balb/c mice (48)	Animal experiment	Enteropathogenic *Escherichia coli*	Able to control the infection.
	Jaiswal et al. [59]	2013	India	Diarrhea	New Zealand white rabbits (6)	Animal experiment	*Vibrio cholerae*	Lowered the shedding of bacteria significantly
	Nale et al. [60]	2016	United Kingdom	Diarrhea	Hamster (NA)	Animal experiment	*Clostridium difficile*	Reduced *Clostridium difficile* colonization at 36 h postinfection.
	Galtier et al. [61]	2016	France	Uropathogenic *Escherichia coli* infection	Balb/cYJ mice (5)	Animal experiment	Uropathogenic *Escherichia coli*	Microbiota diversity was much less affected by phages than by antibiotics and efficiently target uropathogenic *Escherichia coli* strains residing in the gut.

Respiratory infection	Cao et al. [62]	2015	China	Pneumonia	Swiss Webster mice (20)	Animal experiment	*Klebsiella pneumoniae*	Phage-treated mice exhibited a lower level of *Klebsiella pneumoniae* burden in the lungs as compared to the untreated control. These mice lost less body weight and exhibited lower levels of inflammatory cytokines in their lungs.
	Alemayehu et al. [63]	2012	Ireland	Pneumonia and cystic fibrosis	Balb/c mice (16)	Animal experiment	*Pseudomonas aeruginosa*	Effective in killing the pathogen in murine lungs. *Pseudomonas* was effectively cleared from murine lungs in 6 h.
	Oduor et al. [64]	2016	Kenya	Haematogenous *Staphylococcus aureus* pneumonia	Balb/c mice (30)	Animal experiment	*Staphylococcus aureus*	Histology showed that the mice treated with phage did not develop pneumonia. Phage therapy is effective against haematogenous infection.
	Waters et al. [65]	2017	United Kingdom	Chronic lung infections	Balb/c mice (60)	Animal experiment	*Pseudomonas aeruginosa*	Phage therapy was again highly effective against the established 6 d lung infection, completely clearing bacteria from the lungs of 70% of mice and significantly reducing CFU counts in the other 30% compared with controls.
	Bao et al. [41]	2020	China	Recurrent urinary tract infection	Human (1)	Case report	*Klebsiella pneumoniae*	The combination of sulfamethoxazole-trimethoprim with the phage cocktail inhibited the emergence of phage resistant mutant in vitro, and the urinary tract infection of the patient was successfully cured by this combination.

Urinary tract infection	Leitner et al. [66]	2021	Switzerland	Infection after transurethral resection of the prostate	Human (113)	RCT	*Enterococcus* spp., *Escherichia coli*, *Proteus mirabilis*, *Pseudomonas aeruginosa*, *Staphylococcus* spp., and *Streptococcus* spp.	The efficacy of the phage group was similar to that the of antibiotic group (bacterial titer decreased significantly), but the adverse reactions were less.
	Kuipers et al. [67]	2019	Netherlands	Recurrent urinary tract infection after posttransplant	Human (1)	Case report	*Klebsiella pneumoniae*	The infection eventually evolved into epididymitis which was successfully treated with meropenem and phages.
	Rostkowska et al. [68]	2021	Poland	Chronic urinary tract infection after kidney transplantation (caused by polycystic kidney disease)	Human (1)	Case report	*Klebsiella pneumoniae*	Fully recovered following a nephrectomy of his own left kidney.

Eye infection	Fukuda et al. [69]	2012	Japan	Keratitis	C57BL/6 mice (NA)	Animal experiment	*Pseudomonas aeruginosa*	Significantly improved disease outcome and preserved the structural integrity and transparency of the infected cornea. Suppression of neutrophil infiltration and greatly enhanced bacterial clearance in the infected cornea.
	Furusawa et al. [70]	2016	Japan	Keratitis	C57BL/7 mice (NA)	Animal experiment	*Pseudomonas aeruginosa*	A great reduction of bacterial proliferation was shown in phage therapy for mouse models of *Pseudomonas aeruginosa* keratitis (suppressed bacterial multiplication to 0.004%).

Ear infection	Wright et al. [71]	2009	United Kingdom	Chronic otitis	Human (24)	RCT	*Pseudomonas aeruginosa*	No adverse events. Phage-treated group *Pseudomonas aeruginosa* counts were significantly lower only in the phage-treated group.
	Hawkins et al. [72]	2010	United Kingdom	Otitis	Dogs (13)	Animal experiment	*Pseudomonas aeruginosa*	48 h after treatment, the clinical score and *Pseudomonas aeruginosa* count of all ears had fallen.

Nasal infection	Ooi et al. [73]	2019	Australia	Chronic rhinosinusitis	Human (9)	Single arm study	*Staphylococcus aureus*	Preliminary efficacy results indicated favorable outcomes across all cohorts, with 2 of 9 patients showing clinical and microbiological evidence of eradication of infection.

Sepsis/Bacteremia	Schneider et al. [74]	2018	Hungary	Sepsis	Balb/c mice (36)	Animal experiment	*Escherichia coli*	Phage particles administered 10 and 60 min following the bacterial challenge elicited 100% and 95% survival, respectively. But no mice could be rescued if phage administration occurred 3 hours postinfection.
	Pouillot et al. [75]	2012	France	Sepsis and meningitis	Sprague Dawley rat pups (50)	Animal experiment	*Escherichia coli*	When phages were given at 7 h and 24 h after bacterial injection, the survival rates of rats were 100% and 50%, respectively

Novel coronavirus pneumonia	Li et al. [76]	2020	United States	SARS-CoV-2 infection	BALB/c mice (55); Hamster (10)	Animal experiment	SARS-CoV-2	Potently neutralized mouse-adapted SARS-CoV-2 in wild-type mice at a dose as low as 2 mg/kg and exhibited high prophylactic and therapeutic efficacy in a hamster model of SARS-CoV-2 infection

*Note.* NA, not applicable; RCT, randomized controlled trial.

**Table 3:** Clinical therapeutic trials involving the use of phage or phage lytic enzymes.

NCT number	Registry date	Conditions	Interventions	Phases	Locations
NCT00663091	22-April-2008	Venous leg ulcers	Phages cocktail	Phase 1	United States
NCT00937274	10-July-2009	Diarrhea	Phages cocktail	Not applicable	Bangladesh
NCT00945087	23-July-2009	Bacterial infections	Single phage/phages cocktail Phage lytic enzymes	Not applicable	Poland
NCT01617122	12-June-2012	Primary immune deficiency diseases	Single phage	Not applicable	United States
NCT01746654	11-December-2012	Infectious disease/bacterial infections	Phage lytic enzymes	Phase 1/phase 2	Singapore
NCT01818206	26-March-2013	Cystic fibrosis	Phages cocktail	Not applicable	France
NCT01855048	16-May-2013	Healthy volunteers/antibacterial agents	Phage lytic enzymes	Phase 1	Korea
NCT02116010	16-April-2014	Wound infection	Phages cocktail	Phase 1/phase 2	Belgium
NCT02439359	8-May-2015	Bloodstream infections	Phage lytic enzymes	Phase 1	United States
NCT02664740	27-January-2016	Diabetes/diabetic foot	Phages cocktail	Phase 1/phase 2	France
NCT02757755	2-May-2016	Healthy volunteers	Phages cocktail	Phase 1	United States
NCT02840955	21-July-2016	Atopic dermatitis	Phage lytic enzymes	Not applicable	Netherlands
NCT03089697	24-March-2017	Antibacterial agents	Phage lytic enzymes	Phase 2	Korea
NCT03140085	4-May-2017	Urinary tract infections	Phages cocktail	Phase 2/phase 3	Georgia
NCT03163446	23-May-2017	Antibacterial agents	Phage lytic enzymes	Phase 2	United States
NCT03269617	1-September-2017	Gastrointestinal disorder	Phages cocktail	Not applicable	United States
NCT03808103	17-January-2019	Crohn disease	Phages cocktail	Phase 1/phase 2	United States
NCT04191148	9-December-2019	Urinary tract infections	Phages cocktail	Phase 1	United States
NCT04287478	27-February-2020	Urinary tract infection, bacterial	Single phage/phages cocktail	Phase 1/phase 2	United States
NCT04289948	28-February-2020	Diabetes/diabetic foot	Phages cocktail	Phase 1/phase 2	United Kingdom
NCT04323475	26-March-2020	Wound infection	Phages cocktail	Phase 1	Australia
NCT04325685	27-March-2020	Trauma injury/brain injuries Abdominal sepsis/pancreatitis Meningitis/encephalitis/seizures Acute respiratory distress syndrome	Phages cocktail	Not applicable	Russian
NCT04596319	20-October-2020	Cystic fibrosis/lung infection	Phages cocktail	Phase 1/phase 2	United States
NCT04650607	2-December-2020	Prosthetic joint infection/bone and joint infection/implant infection	Single phage/phages cocktail	Not applicable	France
NCT04682964	24-December-2020	Acute tonsillitis	Single phage	Phase 3	Uzbekistan
NCT04684641	24-December-2020	Cystic fibrosis	Single phage	Phase 1/phase 2	United States
NCT04724603	12-January-2021	Bone and joint infection/prosthetic joint infection	Single phage/phages cocktail	Not applicable	France
NCT04737876	4-February-2021	Healthy volunteers	Phages cocktail	Phase 1	United States
NCT04787250	8-March-2021	Prosthetic joint infection	Single phage/phages cocktail	Phase 1/phase 2	United States
NCT04803708	18-March-2021	Diabetic foot ulcer/*Pseudomonas aeruginosa*infection/*Staphylococcus aureus* infection/*Acinetobacter* infection	Phages cocktail	Phase 1/phase 2	Israel
NCT04815798	25-March-2021	Pressure ulcer	Phages cocktail	Phase 1/phase 2	United Kingdom
NCT05010577	18-August-2021	Chronic *Pseudomonas aeruginosa* infection/cystic fibrosis	Phages cocktail	Phase 1/phase 2	Israel
NCT05177107	4-January-2022	Osteomyelitis/diabetic foot osteomyelitis	Single phage/phages cocktail	Phase 2	United States
NCT05182749	10-January-2022	Shigellosis	Phages cocktail	Phase 1/phase 2	United States
NCT05184764	11-January-2022	Bacteremia/*Staphylococcus aureus/Staphylococcusaureus* bacteremia/bacteremia due to *Staphylococcus aureus*	Phages cocktail	Phase 1/phase 2	United States
NCT05240300	15-February-2022	Atopic dermatitis	Single phage/phages cocktail	Phase 1/phase 2	Israel
NCT05269121	7-March-2022	Prosthetic joint infection/bacterial infections	Single phage/phages cocktail	Phase 1/phase 2	United States
NCT05269134	7-March-2022	Prosthetic joint infection	Single phage/phages cocktail	Phase 2/phase 3	United States
NCT05272566	9-March-2022	Feeding patterns/microbial colonization	Fecal phages transfer	Not applicable	Denmark
NCT05272579	9-March-2022	Necrotizing enterocolitis/microbial substitution	Fecal phages transfer	Early phase 1	Denmark
NCT05277350	14-March-2022	*E. coli* infections/bloodstream infection	Phages cocktail	Phase 1	Denmark

Data from https://clinicaltrials.gov.

## Data Availability

The data used to support the findings of this study are available from the corresponding author upon request.

## Abbreviations

VLPs: Virus-like particles
ANI: Average nucleotide identity
ICTV: International Committee on Taxonomy of Viruses
PT: Phylogenetic tree
GSNs: Gene-sharing networks
PAMPs: Pathogen-associated molecular patterns
IgA: Immunoglobulin A
EPEC: Enteropathogenic *Escherichia coli*
SARS-CoV-2: Severe acute respiratory syndrome coronavirus 2
PDT: Phage display technology.
